# Spatiotemporal evolution and influencing factors of agricultural carbon emissions in China

**DOI:** 10.1371/journal.pone.0323824

**Published:** 2025-10-31

**Authors:** Minghui Wei, Mengxue Cao, Dongmei Yin, Fei Li, Yan Lv, Laijun Lu

**Affiliations:** 1 Geomathematics Key Laboratory of Sichuan Province, Chengdu University of Technology, Chengdu, China; 2 School of Mathematical sciences, Chengdu University of Technology, Chengdu, China; 3 School of Public Policy and Administration, Xi’an Jiaotong University, Xi’an, China; 4 College of Information Technology, Jilin Agricultural University, Changchun, China; 5 College of Earth Sciences, Jilin University, Changchun, China; University of Kalyani, INDIA

## Abstract

Clarifying the spatiotemporal characteristics of agricultural carbon emissions and influencing factors in China is crucial. A system for measuring agricultural carbon emissions was established, thus evaluating the level of carbon emissions in China and its provinces. Moreover, the dynamic evolution of agricultural carbon emissions in China and the regions on both sides of the Hu Line was analyzed, then investigated factors affecting agricultural carbon emissions by the LMDI model. The results indicate that the total amount and intensity of agricultural carbon emissions showed an upward and then a downward trend in China from 2001 to 2021. The peaks were 330.72 million tons and 1.98 tons\ha, respectively. Agricultural carbon intensity in provinces was mostly Low-Low Cluster and the range of High-High Cluster has decreased. Inter-provincial disparities in agricultural carbon emissions were also gradually narrowing. These show that the effect of agricultural carbon emissions reduction was obvious in China. It is important to note that carbon emissions from energy consumption in agriculture and agricultural material inputs were substantial, accounting for about 95% of the total. Agricultural carbon emissions were restricted by the agricultural production efficiency, changes in industrial structure, rural population size, and agricultural industrial structure, but were promoted by the level of economy and urbanization. Therefore, we recommend enhancing inter-provincial synergistic collaboration to create agricultural carbon emissions reduction pathways with unique features. It is also essential to maximize agricultural production efficiency and grasp the direction of green and low-carbon. We also suggest that the Chinese government should accelerate the in-depth adjustment and transformation and upgrading of the industrial structure, thereby reducing agricultural carbon emissions at source.

## 1. Introduction

Global climate change is a significant challenge shared by all countries because it is profoundly affecting human survival and development. Some studies have shown that greenhouse gas (GHG) emissions from human activities have accelerated global warming. The Food and Agriculture Organization (FAO) has emphasized that the food system is to blame for more than one-third of all GHG emissions [1]. In order to counteract climate change and encourage the sustainable development of human society, the Intergovernmental Panel on Climate Change (IPCC) has recommended that global carbon emissions peaks as early as possible during 2020–2030 and to carbon neutrality by 2050. China has resolutely reacted to the call by pledging to achieve a carbon peak by 2030 and carbon neutrality by 2060, aiming to support the green transformation of economic structure and the creation of a low-carbon and energy-saving industrial system. It is generally known that agriculture not only is the second largest source of GHG emissions but also contributes to the greenhouse effect on a large spatial and temporal scale [2]. As an agricultural country, China has great potential in terms of reducing agricultural carbon emissions. In addition, China has clearly pointed out the crucial significance of agricultural carbon reduction and sequestration in the Implementation Plan of Agricultural and Rural carbon reduction and sequestration in 2022. Therefore, it is of great significance to study the spatiotemporal characteristics and factors of agricultural carbon emissions in China, thus effectively responding to climate change, optimizing and adjusting the industrial structure, while better promoting rural revitalization and the construction of the agricultural ecological civilization.

Carbon emissions from agriculture are GHG emissions caused by agriculture, which primarily include carbon dioxide, methane, and nitrous oxide [3]. From the perspective of domestic and foreign studies, the researchers on agricultural carbon emissions have focused on the measurement, spatial and temporal evolution characteristics and influencing factors [4,5]. In terms of measuring agricultural carbon emissions, researchers mainly utilized the carbon emission factor method to quantify agricultural carbon emissions from the standpoint of production and management. The system of measurement included indicators for fertilizers, pesticides, diesel and other indicators from agricultural production and management [6, 7–9], involving five aspects of agricultural materials use, rice growth, land management, livestock and poultry breeding, and energy consumption [10,11–13]. However, the measurement index system was not complete.

Based on the measurement results of agricultural carbon emissions, scholars have mostly analyzed the temporal characteristics and spatial change trends with agricultural carbon intensity (ACI) as an indicator, thus revealing the evolution of the spatiotemporal patterns of agricultural carbon emissions in the study area and influencing factors [14–16]. Currently, the research on the spatiotemporal patterns and spatial heterogeneity of agricultural carbon emissions is relatively weak, namely, the visualization technology of spatial autocorrelation has not been fully utilized and there is also a lack of analysis of distribution dynamics from a new perspective. Meanwhile, researchers have focused on dynamic quantitative analysis from the socio-economic and within the agricultural system when analyzing the influencing factors of agricultural carbon emission [17–19]. For example, Guo et al. used models such as the causality test and double fixed effect regression to discuss the impact of factors such as fiscal input for agriculture, green finance, and farmland transfer on agricultural carbon emissions [20–22]. In addition, scholars have constructed the Log-Mean Divisia Index (LMDI) model from the three levels of economic production, industrial structure, and demographic factors, thus analyzing the relationship between factors such as agricultural industrial structure and urbanization and agricultural carbon emissions [23–25]. Especially, the LMDI model was very effective due to the advantages of residual-free decomposition and strong applicability [26–28]. However, most of the studies only focused on a certain agricultural sector or region and lacked an examination of factors in China as a whole.

As can be seen from the literature combined above, the existing research on agricultural carbon emissions has provided many valuable theoretical results, but there are some shortcomings. In order to fill the above research gaps, the contribution of this paper is mainly reflected in the following three aspects. First, a system was built to quantify the overall amount and intensity of agricultural carbon emissions in the provinces of China from 2001 to 2021, thus investigating the temporal and spatial patterns of agricultural carbon emissions. Second, quantitative analysis for the spatial autocorrelation of agricultural carbon emissions on an inter-provincial scale was done with the help of the Local Indicators of Spatial Association (LISA) agglomeration map by using ACI as an indicator. Meanwhile, the Hu Line was not only a transition zone of climatic conditions but also a paramount demarcation line of the coupling characteristics of food production and agricultural labor force changes [29,30]. Therefore, with the new perspective of Hu Line, the inter-provincial disparity of ACI and its dynamic evolution characteristics in China and both sides of Hu Line was analyzed by using the Kernel density. Finally, the Log-Mean Divisia Index (LMDI) model was built from the economic, social, and demographic levels to study the factors influencing agricultural carbon emissions in China, thus, some policy implications for the development of low-carbon agriculture and the promotion of agricultural structure transformation and upgrading in China was provided.

## 2. Materials and methods

### 2.1. Measurement of agricultural carbon emissions

Synthesizing the research of several scholars, we used the carbon emission factor method to measure agricultural carbon emissions from five aspects (Table 1) and referred to the IPCC Fourth Assessment Report to convert all types of GHG into standard carbon dioxide, with the following formula:

**Table 1:** Sources of GHG emissions from agriculture.

Measurement dimensions	Sources	Indicators
Agricultural inputs materials	Fertilizer	Amount of nitrogen fertilizer used (kg) Amount of phosphorus fertilizer used (kg) Amount of potash fertilizer used (kg) Amount of compound fertilizer used (kg)
	Pesticides	Amount of pesticide used (kg)
	Agricultural film	Amount of film used (kg) Amount of agricultural plastic film used (kg)
Energy consumption in agriculture	Diesel fuel	Amount of agricultural diesel fuel used (kg)
	Electricity	Amount of rural electricity used (kw·h)
Land management	Land plowing	Crop Sowing Area (hm^2^)
	Irrigation	Effective irrigation area (hm^2^)
Cultivation of rice	Early, middle and late rice	Cultivation area of early, middle and late rice (hm^2^)
Livestock breeding	Enteric fermentation	Average annual rearing capacity of cattle, sheep, pigs (head)
	Manure management	Average annual rearing capacity of cattle, sheep, pigs and poultry (head)


$$E=\sum_{i=\text{1}}^{n}E_{i}A_{i}$$ (1)

Where E represents total agricultural carbon emissions; $E_{i}$ represents carbon source of the i-th; and $A_{i}$ represents the corresponding carbon emission factor. Moreover, the carbon emission coefficient comes from the study of Xiangdong Hu et al. [17,31,32], and the details of the calculations are shown in the Supplementary Material.

Since the carbon intensity can objectively reflect the carbon emission level, we defined ACI as the agricultural carbon emission per unit area [33], which is calculated by the formula:


$$I_{E}=\frac{E}{S}$$ (2)

Where S is the total sown area of crops and $I_{E}$ is the carbon intensity of agriculture in tons/ha.

### 2.2. Spatial autocorrelation analysis

The Moran’s I index plays a crucial role in spatial autocorrelation analysis, in which the Global Moran’s I index determines whether there is agglomeration in the region, and the Local Moran’s I index identifies the spatial heterogeneity through the LISA agglomeration map [34]. The global Moran’s I index used in this paper was calculated as follows:


$$I=\frac{N}{\sum_{i=\text{1}}^{N}\sum_{j=\text{1}}^{N}W(i,j)}\cdot \frac{\sum_{i=\text{1}}^{N}\sum_{j=\text{1}}^{N}W(i,j)(C_{i}-\overset{―}{C})(C_{j}-\overset{―}{C})}{\sum_{j=\text{1}}^{N}{\left(C_{i}-\overset{―}{C}\right)}^{\text{2}}}$$ (3)

The Local Moran’s I index is as follows:


$$I_{i}=\frac{C_{i}-\overset{―}{C}}{\sqrt{\sum_{i=\text{1}}^{N}\left(C_{i}-\overset{―}{C}\right)/(N-\text{1})}}\cdot \sum_{j=\text{1}}^{N}W(i,j)(C_{j}-\overset{―}{C})$$ (4)

Where N is the number of provinces; $C_{i}$ is ACI of the i-th province; $\overset{―}{C}$ represents the average value of ACI in each province; and W(i,j) represents the spatial connectivity matrix between provinces i and j.

### 2.3. The kernel density method

**Fig 1:**
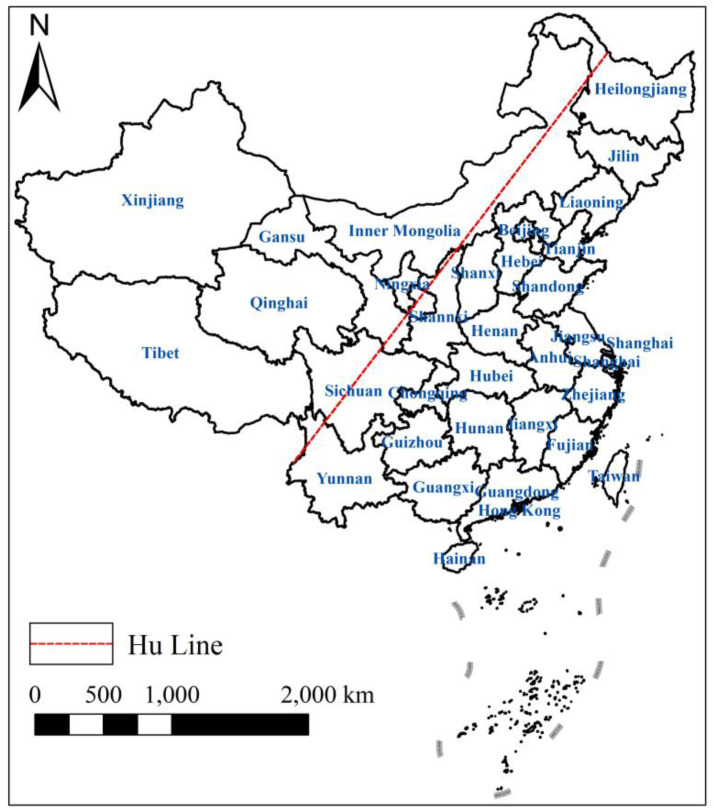
Hu Line. Note: This map is based on the standard map with the review number GS(2023)2763 downloaded from the Standard Map Service website of the Ministry of Natural Resources of the People’s Republic of China. The base map has not been modified.


The regions on both sides of the Hu Line were divided into the southeastern side and the northwestern side based on the provincial boundary [35] (Fig 1). In order to clarify the distributional dynamics and evolutionary patterns of the absolute differences in ACI across the country and both sides of Hu Line, the kinetics, extensibility, and polarization of the distribution of ACI were examined using the Gaussian kernel function [36]. The density function of ACI is according to Eq. (5). Where *N* is the number of observed values; $X_{i}$ represents independent and equally distributed observations; x represents the mean of the observations; *h* is the bandwidth; K(x) is the Gaussian kernel function, see Eq. (6).

$$f(x)=\frac{\text{1}}{Nh}\sum_{i}^{N}K(\frac{X_{i}-x}{h})$$ (5)

$$K(x)=\frac{\text{1}}{\sqrt{\text{2}\pi }}\text{exp}(-\frac{x^{\text{2}}}{\text{2}})$$ (6)

### 2.4. The LMDI model

From the standpoint of social, economic and population factors, the agricultural production efficiency $\left(F_{\text{1}}\right)$, agricultural industrial structure $\left(F_{\text{2}}\right)$, changes in industrial structure ${(F}_{\text{3}})$, economic level $\left(F_{\text{4}}\right)$, urbanization level ${(F}_{\text{5}})$ and rural population size$\;{(F}_{\text{6}})$ were used as influencing factors to construct the following LMDI model of agricultural carbon emissions in China.


$$E=F_{\text{1}}\cdot F_{\text{2}}\cdot F_{\text{3}}\cdot F_{\text{4}}\cdot F_{\text{5}}\cdot F_{\text{6}}$$ (7)

where $F_{\text{1}}=E/x_{\text{1}}$, $x_{\text{1}}$ denotes the gross value of agriculture and livestock; $F_{\text{2}}=x_{\text{1}}/x_{\text{2}}$,$\;x_{\text{2}}$ denotes the gross value of agriculture, forestry, livestock and fisheries; $F_{\text{3}}=x_{\text{2}}/x_{\text{3}}$, $x_{\text{3}}$ denotes the gross value of the regional product;$F_{\text{4}}=x_{\text{3}}/x_{\text{4}}$, $x_{\text{4}}$ denotes the total population of the region; $F_{\text{5}}=x_{\text{4}}/x_{\text{5}}$, $x_{\text{5}}$ denotes rural population of the region; and $F_{\text{6}}=x_{\text{5}}$. $F_{\text{1}}$ and $F_{\text{4}}$ are in tons/million and million dollars/person, respectively.

The LMDI addition and decomposition was used to quantify the influence of each factor on carbon emissions, specifically, as follows:


$$\Delta E=E_{t}-E_{\text{0}}=\sum_{w=\text{1}}^{\text{6}}{\Delta F}_{w}$$ (8)

$${\Delta F}_{w}=\sum \frac{E_{t}-E_{\text{0}}}{lnE_{t}-{lnE}_{\text{0}}}\cdot (lnF_{w}^{t}-lnF_{w}^{\text{0}})$$ (9)

where ΔE is the total effect; t denotes the period (t=1,2,...,T); 0 denotes the base period; and ${\Delta F}_{w}$ denotes the contribution of the factor $F_{w}$ to the amount of changes in agricultural carbon emissions. If the amount of carbon emission change caused by the factor is positive, it means that the factor presents a positive effect on carbon emission and plays a role of promotion. If it causes a negative value, it presents a negative effect and plays a role of inhibition.

### 2.5. Data sources

The data about measurement of agricultural carbon emissions and intensity mainly were from China Rural Statistical Yearbook, China Agricultural Statistical Yearbook, China Animal Husbandry, and Veterinary Yearbook and China Provincial Statistics Yearbook. Agricultural material inputs and energy consumption, land management, and cultivation of rice were all based on the actual value of the Statistical Yearbook. Meanwhile, the calculation of livestock and poultry breeding quantity was combined with the number of livestock and breeding cycle at the end of the year, in which the life cycle of pigs, sheep, cattle and poultry was 200d, 365d, 210d and 55d respectively [37]. In addition, individual missing data involved in land management were supplemented by the moving average method. Besides, the data to explore the factors affecting agricultural carbon emissions came from the China Rural Statistical Yearbook and the China Statistical Yearbook. The datasets used in this study have been deposited in the figshare (https://doi.org/10.6084/m9.figshare.29967226).

## 3. Analysis of empirical results

### 3.1. Spatiotemporal patterns of agricultural carbon emissions

#### 3.1.1. Timing characteristics.

**Fig 2:**
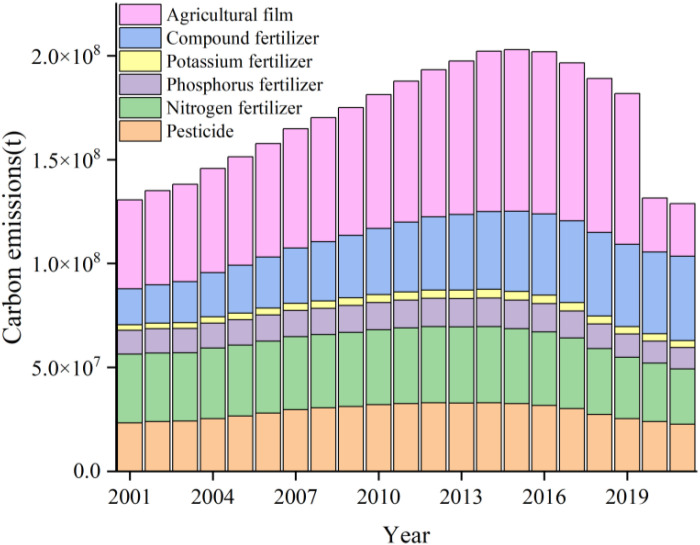
Structure of carbon emissions from agricultural material inputs.

It can be seen from Table 2 that the structure of agricultural carbon emissions in China was quite stable, and agricultural material inputs and agricultural energy consumption always played a dominant role, followed by land management, while rice planting and livestock breeding accounted for a relatively small proportion. Longitudinally, agricultural carbon emissions totaled 234.872 million tons in China in 2021, with an increase of 7.18% compared with 2001 and an average annual increase of 0.53%. ACI in 2021 was 1.39 tons per hectare, which was 0.49% lower than that in 2001. In addition, there was a significant downward trend in carbon emissions from livestock farming alone, with a cumulative decrease of 16.88%. Based on the carbon emissions generated by the various subsystems of agricultural material inputs (Fig 2), it is clear that agricultural film was the primary source of carbon in agricultural material inputs, followed by pesticides, compound fertilizers, nitrogen fertilizers and phosphate fertilizers.

**Table 2:** Structure, total amount and intensity of agricultural carbon emissions in China from 2001 to 2021.

Year	Percentage (%)					Total volume (tons)	Intensity (tons/ha)
	$S_{1}$	$S_{2}$	$S_{3}$	$S_{4}$	$S_{5}$		
2001	59.97	36.82	3.13	0.07	0.02	21786.5	1.40
2002	60.44	36.44	3.04	0.06	0.02	22347.8	1.45
2003	60.01	36.97	2.94	0.06	0.02	23015.0	1.51
2004	58.04	39.17	2.72	0.06	0.02	25101.6	1.63
2005	57.93	39.36	2.64	0.06	0.02	26121.0	1.68
2006	58.69	38.66	2.57	0.05	0.02	26877.2	1.77
2007	58.61	38.84	2.49	0.05	0.01	28122.6	1.83
2008	60.85	36.51	2.58	0.05	0.01	27965.0	1.79
2009	60.67	36.73	2.54	0.05	0.01	28854.7	1.82
2010	60.76	36.69	2.49	0.05	0.01	29827.8	1.86
2011	61.19	36.28	2.47	0.05	0.01	30680.6	1.89
2012	61.31	36.19	2.43	0.05	0.01	31516.7	1.93
2013	61.31	36.21	2.41	0.05	0.01	32204.6	1.96
2014	61.61	35.92	2.40	0.05	0.01	32798.7	1.98
2015	61.47	36.04	2.43	0.05	0.01	33007.2	1.98
2016	62.15	35.28	2.51	0.05	0.01	32488.1	1.95
2017	61.73	35.64	2.57	0.05	0.01	31835.6	1.91
2018	61.79	35.46	2.69	0.05	0.01	30600.1	1.84
2019	61.62	35.52	2.80	0.05	0.01	29498.4	1.78
2020	54.75	41.70	3.47	0.06	0.01	24023.1	1.43
2021	54.87	41.51	3.54	0.06	0.01	23487.2	1.39
Average growth rate (%)	0.22	1.09	1.00	0.22	-0.79	0.53	0.13
Cumulative Growth rate (%)	-1.36	21.56	21.98	4.03	-16.88	7.18	-0.49

*Notes*: $S_{\text{1}}$ to $S_{\text{5}}$ represent agricultural material inputs, agricultural energy consumption, land management, cultivation of rice and livestock breeding, respectively.

**Fig 3:**
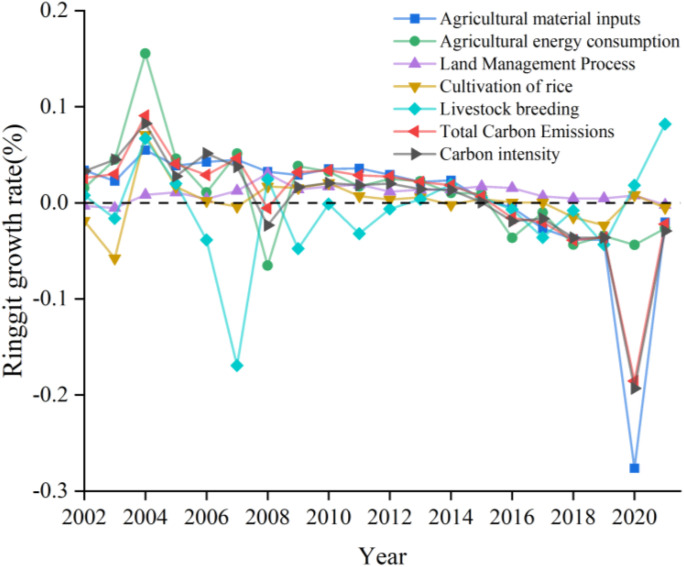
Changes in the growth rate of agricultural carbon emissions in China.

At the same time, there were certain inter-annual fluctuations in the total amount and intensity of agricultural carbon emissions in China from 2001 to 2021, which can be roughly divided into three stages (Fig 3): fluctuation rise, keep rising and fluctuation decline. From 2001 to 2010 (period P1), with a large increase in the total amount and intensity of agricultural carbon emissions. From 2010 to 2015 (period P2), the growth rate of the total amount and intensity fluctuated slightly, among which the carbon emission caused by the input of agricultural materials in 2015 was as high as 61.47% of the total. Evidently, the increase of agricultural material inputs and agricultural energy consumption were the main reasons in the two stages of the rise. From 2016 to 2021 (period P3), in which the total amount and intensity of agricultural carbon emissions began to decline, with the largest negative growth rate of 18.56% in 2020. Causes of this phenomenon may be the support of Chinese strategies, such as high-quality development, the effective utilization of agricultural materials and energy inputs.

#### 3.1.2. Spatial differences.

It is easy to find that ACI passed the test in most years (Tables 3). Furthermore, ACI showed a concentrated distribution during the period P1 (2001–2010) and the period P2 (2011–2015). However, during the period P3 (2015–2021), the random distribution and the agglomeration distribution appeared alternately.

**Table 3:** The global Moran’s index of ACI during the period P1 (2001-2010).

Year	2001	2002	2003	2004	2005	2006	2007	2008	2009	2010
Index	0.215	0.288	0.295	0.349	0.337	0.274	0.273	0.222	0.195	0.179
Test results	***	***	***	***	***	***	***	***	***	***

***N****otes*: *** represent significance at 1% level.

**Table 4:** The global Moran’s index of ACI during the period P2 (2011-2015).

Year	2011	2012	2013	2014	2015
Index	0.144	0.144	0.147	0.143	0.141
Test results	**	**	**	**	**

*Notes*: ** represent significance at 5% level.

**Table 5:** The global Moran’s index of ACI during the period P3 (2015-2021).

Year	2016	2017	2018	2019	2020	2021
Index	0.097	0.105	0.079	0.073	0.112	0.023
Test results	*	*			**	

*Notes*: *, ** represent significance at 10%, and 5% level respectively.

**Fig 4:**
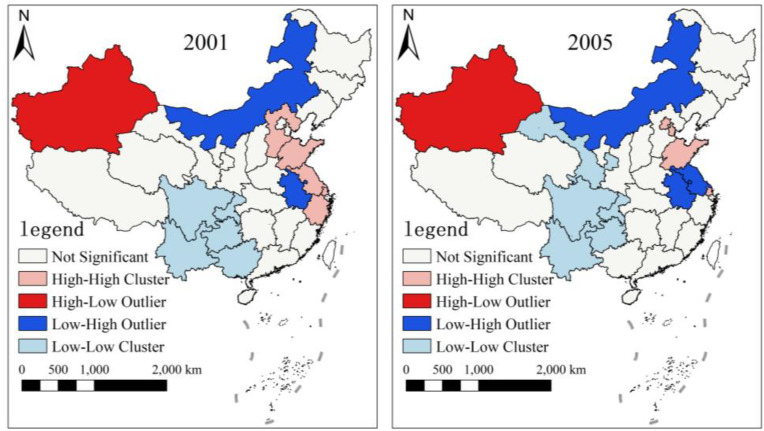
Spatial agglomeration maps of ACI in 2001 and 2005. Note: This map is based on the standard map with the review number GS(2023)2763 downloaded from the Standard Map Service website of the Ministry of Natural Resources of the People’s Republic of China. The base map has not been modified.

In order to clearly reflect the spatial pattern of agricultural carbon emissions in China, we drew the LISA agglomeration maps representing six time nodes, namely, 2001, 2005, 2010, 2015, 2020 and 2021, based on ACI of 31 provinces in China (Figs 4^6^). The changes in the clustering pattern of ACI show that compared with 2001, the High-High Cluster in 2005 was mainly around the Bohai Sea, which may be due to the fact that Shandong and Henan were large agricultural provinces with high consumption of agricultural input materials and energy, and thus higher ACI. The Low-Low Cluster was in Southwest China, probably because Southwest China had a simpler agricultural industrial structure and invested less in agricultural materials compared to neighboring provinces, so ACI was lower. In addition, High-Low Outlier was exhibiting around Xinjiang, which may be related to the cultivated area and the level of agricultural technology. Compared with 2005, the range of High-High Cluster significantly reduced in 2010. Compared to 2015, the range of High-Low Outlier also decreased in 2020, while the range of Low-High Outlier increased. By 2021, the range of Low-High Outlier decreased compared with the previous year, and there were no provinces with High-High Cluster. The possible cause of these changes was that with the promotion of national resource conservation and environmentally friendly policies, regions with higher ACI had achieved more obvious carbon emission reduction effects. In addition, Shanxi was also determined to have a High-Low Outlier, which could be employed as a focus item in the future.

**Fig 5:**
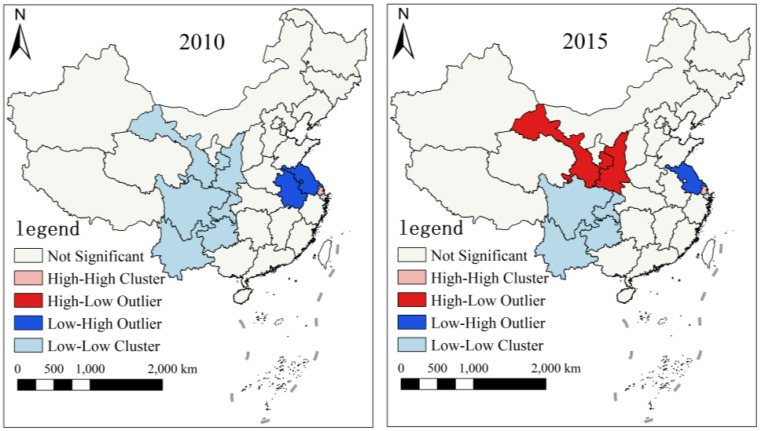
Spatial agglomeration maps of ACI in 2010 and 2015. Note: This map is based on the standard map with the review number GS(2023)2763 downloaded from the Standard Map Service website of the Ministry of Natural Resources of the People’s Republic of China. The base map has not been modified.

**Fig 6:**
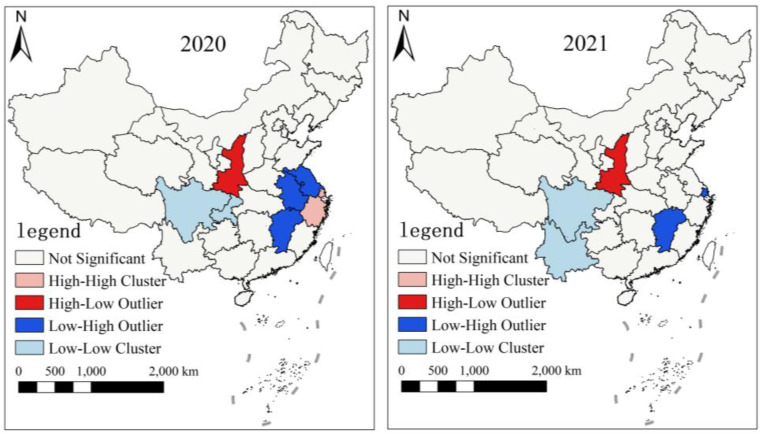
Spatial agglomeration maps of ACI in 2020 and 2021. Note: This map is based on the standard map with the review number GS(2023)2763 downloaded from the Standard Map Service website of the Ministry of Natural Resources of the People’s Republic of China. The base map has not been modified.

#### 3.1.3. Dynamic evolution.

**Fig 7:**
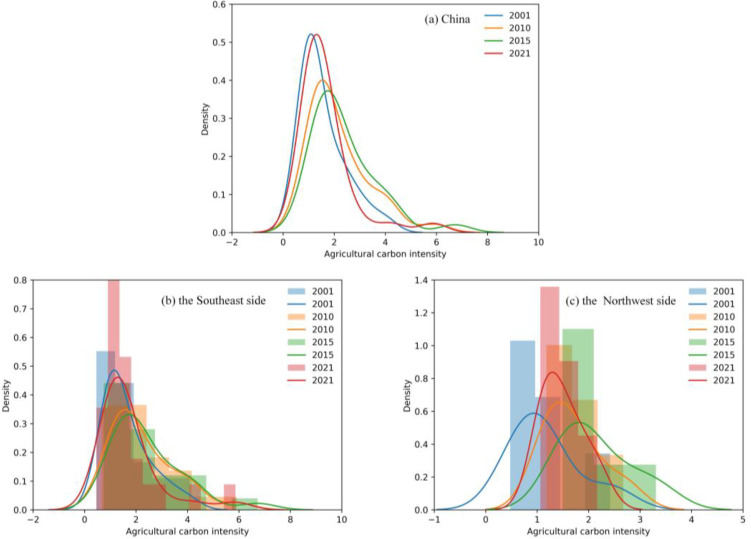
Results of Kernel density of ACI in China, the southeastern side and the northwestern side.

According to the characteristics of inter-annual fluctuations of agricultural carbon emissions in section 3.1.1, we used the Kernel density mentioned in section 2.3 to investigate the evolution characteristics and distribution dynamics of ACI in China and both sides of Hu Line at four-time points (2001, 2010, 2015, 2021) in three stages (Fig 7).

From the distribution curve of ACI in China (Fig 7(a)), we can see that the major peak’s position shifts first to the right and then to the left, while the entire image shifts to the right. Additionally, the height of the major peak is decreasing and then increasing, but generally decreasing. There is only one primary peak without polarization. Compared with 2001, the curve shape of 2010 changes greatly, which is reflected in the curve shifting to the right, the peak value decreasing and the change range expanding. This indicates that ACI increased during the period P1 (2001–2010) and the inter-provincial gap widened. Although the shape of the curve in 2015 is similar to that in 2010, the peak of the curve decreases and the position shifts to the right in 2015, which shows that ACI increased during the period P2 (2011–2015), and a provincial gap continued to expand. Compared with 2015, the distribution curve of ACI in 2021 shifts to the left as a whole, the peak value increases and the width Narrows. These changes indicate that the ACI decreased and the differences among provinces became smaller. Compared with 2001 in China, during the period P3 (2015–2021), there was still an imbalance in ACI across the country in 2021. This may be because different functional attribute positioning in each province led to differences in the selection of agricultural development strategies and models, thus affected the adjustment of industrial structure and the promotion of agricultural modernization, and objectively led to the inter-provincial gap in ACI.

From the overall movement of the distribution curve and the change of the vertical height of the wave crest (Fig 7 (b)), it can be seen that the trend of the southeastern side of Hu Line is consistent with that in China, as detailed below. First, the position of the main peak shifts first to the right and then to the left, while the peak first decreases and then increases. Second, the right trailing ductility widened and there is always a principal peak. Finally, there is no regional polarization. In general, during the period P1 (2001–2010), ACI in the southeastern side increased on the whole, and the inter-provincial gap first increased and then decreased. Compared with 2001, ACI of the southeastern side increased in 2010 and the inter-provincial gap intensified. In 2015, it still increased and the inter-provincial gap widened. Compared with 2015, the distribution curve of ACI in 2021 shifts significantly to the left, while the peak value increases and the width narrows. This shows that the overall ACI in the region decreased during the period P3 (2015–2021), and the gap between provinces became smaller. This may be due to the fact that the five provinces of Tianjin, Shanghai, Hainan, Hebei and Beijing have seen a larger decline in ACI, thus narrowing the gap with other provinces in the southeastern side of Hu Line.

From the evolution of ACI in the northwestern side of Hu Line (Fig 7(c)), it can be seen that the center change of the distribution curve of the density function is right to left shift, and the overall trend is right to shift with a large amplitude. It is clear that the main peak increases and the width narrows, and there is only one main peak without regional polarization. Compared with 2001, ACI in the northwestern side increased in 2010. Compared with 2010, the center of density function shifts to the right in 2015, indicating a continuous increase in ACI of the northwestern side. Meanwhile, the decrease of the peak value and the expansion of the variation range indicate that the inter-provincial gap expanded. On the whole, where ACI showed an increasing trend before 2015, and a decreasing trend after 2015. During the period P3 (2015–2021), the vertical height of the wave peak increases and the horizontal width becomes narrower, which indicates that the regional difference of ACI in the northwestern side of Hu Line showed a narrowing trend. It may be due to the implementation of the national western development strategy that the economic development level of various provinces was gradually approaching, so the agricultural development level was gradually converging, thus the inter-provincial gap in ACI was also narrowing.

### 3.2.. Factors influencing agricultural carbon emissions

We decomposed agricultural carbon emissions in China by utilizing the LMDI model constructed in subsection 2.4 (Table 6). As a result, over the last 21 years, the level of economy and urbanization have been the primary drivers of the increase in agricultural carbon emissions in China, contributing 631.541 million tons and 209.718 million tons, respectively. However, the agricultural production efficiency, changes in industrial structure, rural population size, and agricultural industrial structure, became the most important factors in reducing agricultural carbon emissions in China, contributing −446.432 million tons, −182.617 million tons, −177.711 million tons, and −17.493 million tons, in that order.

**Table 6:** Decomposition results for changes in total agricultural carbon emissions in China.

Year	Contribution value (tons)						
	$\Delta F_{1}$	$\Delta F_{2}$	$\Delta F_{3}$	$\Delta F_{4}$	$\Delta F_{5}$	$\Delta F_{6}$	C
2001-2002	−363.72	−72.76	−1319.91	2127.62	161.86	28.24	561.33
2002-2003	−303.74	−858.92	−1436.58	3112.11	94.20	60.10	667.17
2003-2004	−3121.24	417.30	337.71	4297.04	27.12	128.73	2086.65
2004-2005	−1097.17	−58.19	−2251.28	4508.88	−259.84	176.96	1019.37
2005-2006	213.10	−355.74	−3232.21	3943.14	7723.99	−7536.09	756.19
2006-2007	−4076.17	354.36	−80.94	4863.94	580.94	−396.71	1245.41
2007-2008	−5091.08	272.41	−253.30	4690.77	640.56	−417.02	−157.67
2009−2009	53.23	−427.76	−1339.09	2386.17	570.44	−353.23	889.76
2009-2010	−3216.80	129.94	−1200.46	5052.98	1188.24	−980.82	973.09
2010-2011	−3973.45	2.09	−517.34	5192.45	848.54	−699.45	852.85
2011-2012	−1958.39	−175.86	−153.98	2743.61	1142.23	−761.58	836.03
2012-2013	−1713.46	−177.71	−245.89	2637.20	768.53	−580.78	687.90
2013-2014	−913.21	−199.71	−981.83	2470.36	830.58	−612.02	594.17
2014-2015	−1209.99	151.02	−529.71	1634.72	869.48	−707.06	208.45
2015-2016	−30.11	−562.25	−2425.14	2284.31	981.67	−767.53	−519.05
2016-2017	−1134.54	−368.32	−1802.35	2473.66	922.07	−743.02	−652.51
2017-2018	−2194.95	−230.32	−1205.55	2276.94	758.90	−640.55	−1235.53
2018-2019	−3955.00	223.71	−7441.75	9965.57	674.24	−568.43	−1101.66
2019-2020	−8733.84	441.09	9049.77	−6271.75	1900.54	−1861.18	−5475.37
2020-2021	−1822.65	−253.69	−1231.82	2764.39	547.53	−539.62	−535.86
Total	−44643.2	−1749.3	−18261.7	63154.1	20971.8	−17771.1	1700.7

*Notes*: (1) $\Delta F_{\text{1}}$ through $\Delta F_{\text{6}}$ are the contributions of the agricultural production efficiency, agricultural industrial structure, changes in industrial structure, economic level, urbanization level and rural population size to the amount of changes in agricultural carbon emissions, in that order. (2) C is the amount of change in agricultural carbon emissions.

**Fig 8:**
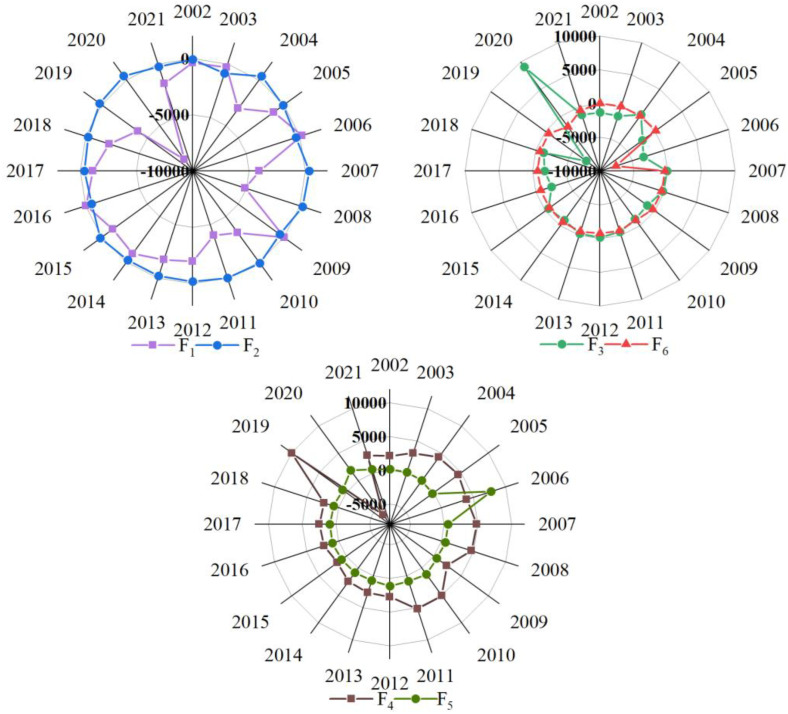
The effects of factors on agricultural carbon emissions. (${𝐅}_{1}$ through ${𝐅}_{6}\;$are the factors of the agricultural production efficiency, agricultural industrial structure, changes in industrial structure, economic level, urbanization level, and rural population size in order.).

According to the inter-annual effects of the six factors on carbon emissions from agriculture in China (Fig 8), it is clear that agricultural production efficiency mainly played an inhibitory role in agricultural carbon emissions. The negative effect presented by the agricultural production efficiency peaked in 2020 at about 87,338,400 tons, which was significantly higher than other factors that play a suppressive role, indicating that the improvement of the productivity in agriculture has effectively suppressed agricultural carbon emissions in recent years. Moreover, with the results of the temporal characteristics of agricultural carbon emissions, it can also be seen that improving the utilization efficiency of agricultural resources is an important measure to reduce agricultural carbon emissions.

The changes in industrial structure and rural population size had a negative impact on agricultural carbon emissions. In particular, the changes in industrial structure presented the most pronounced negative effect in 2019, while presented a positive effect on China’s agricultural carbon emissions for the first time in 2020. Therefore, changes and adjustments in industrial structure deserve attention in the future. In addition, the rural population has been holding down carbon emissions from agriculture in China since 2006. The reason for this phenomenon is that with the improvement of the urbanization level, rural population transfer reduced the scale of the rural labor force, thus restraining agricultural carbon emissions to a certain extent.

The effect of agricultural industrial structure on agricultural carbon emissions had a dynamic fluctuation trend, which indicates that agricultural industrial structure has been changing and adjusting in China, and the proportion of agriculture and animal husbandry in agriculture, forestry, animal husbandry and fishery was unstable. In particular, agricultural industrial structure increased carbon emissions by 4,410,900 tons in 2020 but decreased by 2,536,900 tons in 2021. We can see that it has a direct impact on agricultural carbon emissions. Therefore, it is necessary to continuously optimize the agricultural industrial structure and develop an agricultural industrial system with high output value and low emissions.

The positive effect of the level of economy and urbanization on agricultural carbon emissions changed from 2002 to 2021 (Fig 8). Moreover, the economic level reached the maximum contribution value of 99,655,700 tons in 2019, meanwhile, the urbanization level reached the maximum contribution value of 77.239,900 tons in 2006. Furthermore, the total contribution caused by the economic level is about three times that of the urbanization level. This suggests that the economic level contributes more to China’s agricultural carbon emissions, namely, promoting high-quality economic development will be a helpful plan.

## 4. Conclusions

In this study, the system to measure the total amount and intensity of agricultural carbon emissions in China and its 31 provinces was constructed. The spatial and temporal patterns of agricultural carbon emissions were analyzed and the influencing factors were discussed. The main findings are as follows.

In general, the total amount and intensity of carbon emissions from agriculture showed a trend of first increasing and then decreasing with 2015 as the cut-off point in China from 2001 to 2021. What’s obvious is that China has not only achieved significant carbon emission reduction in the agricultural sector but also made positive progress in the green development of agriculture. It is worth noting that agricultural inputs materials and energy consumption occupied a leading position in agricultural carbon emissions in China, followed by land management, cultivation of rice, and livestock breeding. Therefore, in order to reduce agricultural carbon emissions from the root, we should focus on efficient utilization of agricultural inputs materials and energy inputs, improve the quality of arable land, and cultivate high-quality rice.

At the provincial level, the number of provinces with high ACI had decreased up to 2021, and the inter-provincial gap in ACI on both sides of Hu line had narrowed, which was inseparable from the implementation of the energy-saving and emission reduction strategies. In addition, the inter-provincial gap in the northwestern side was significantly smaller than that in the southeastern side, which was closely related to the economic level and agricultural structure of the southeastern side, implying that the southeastern side has a higher potential for agricultural carbon emission reduction. Therefore, if efficient measures are to be designed, it would be helpful to focus on provinces with agglomeration effects and to incorporate the historical significance of the Hu Line.

While analyzing the factors influencing carbon emissions from agriculture, it was found that agricultural production efficiency, agricultural industrial structure, changes in industrial structure, and rural population size depressed agricultural carbon emissions, but economic level and urbanization level promoted it. Furthermore, the inhibitory effect of agricultural productivity was superior, while the promotional effect of the economic level was greater. Therefore, it can be demonstrated that increasing agricultural production efficiency, as well as optimizing and changing industrial structure, are effective methods to reduce agricultural carbon emissions. Moreover, encouraging economic green development may be viewed as a strategy of minimizing the contribution of the economic level to agricultural carbon emissions.

The above findings of this study reveal the following implications for accelerating the reduction of agricultural carbon emissions. First of all, agricultural materials and energy (such as agricultural film, diesel, etc.) produce more carbon dioxide, so the government should strengthen resource-saving and environmentally friendly agricultural models, form green and low-carbon production methods, and improve agricultural production efficiency. Second, agricultural carbon emissions had obvious agglomeration characteristics among provinces in China, and also there are certain differences in economic level and industrial base. Therefore, it is necessary to strengthen the cooperation between provinces, reasonably change the internal structure of agriculture, forestry, animal husbandry, and fishery industry, and form distinctive agricultural carbon emissions reduction routes. Moreover, with the rapid development of new urbanization in China, to promote the process of agricultural carbon emission reduction, we should not only speed up the deep adjustment of industrial structure but also focus on the transformation and upgrading of agricultural industrial structure and the changing trend of the rural population.

## Supporting information

S1 File Measurement of agricultural carbon emissions. (DOCX)

S1 Data Data. (XLSX)
