# Supplementary material for: Spatiotemporal evolution and influencing factors of agricultural carbon emissions in China
Source: PLoS One. 2025 Oct 31;20(10):e0323824. doi: 10.1371/journal.pone.0323824 (PMC12578253; doi:10.1371/journal.pone.0323824)
Supplement: S1 File — (DOCX) [file pone.0323824.s001.docx]

# S1 File. Measurement of agricultural carbon emissions

The agricultural inputs materials include the carbon and carbon dioxide generated in the production, transportation and use of fertilizers, pesticides and agricultural films. The relevant carbon emissions coefficient is referred to the research of Ziyue Fan, Bo Li and the Institute of Agricultural Resources and Ecological Environment of Nanjing Agricultural University [11,31]. Energy consumption in agriculture mainly refers to diesel and electricity. Where carbon emission coefficients are from IPCC and the National Development and Reform Commission of China. The soil organic carbon loss due to land tillage in land management and the carbon emission from agricultural irrigation, where carbon emission coefficients are taken from IPCC and the School of Biology and Technology of China Agricultural University. In terms of cultivation of rice, in six different parts of China, namely, North China, East China, South Central China, Southwest China, Northeast China, and Northwest China, the amount of methane released by rice during growth was measured. Where carbon emission coefficients are taken from the Guidelines for the preparation of provincial-level greenhouse gas inventories in China (Trial). What’s more, Livestock breeding involves methane and nitrous oxide produced during intestinal fermentation and manure management of pigs, sheep, cattle and poultry. The carbon emission coefficient is derived from the Guidelines for the preparation of provincial-level greenhouse gas inventories in China (Trial) and the research by Xiangdong Hu [31]. Especially, carbon emission coefficient of cattle is taken as the average of dairy cows, buffalo and yellow cattle.
